# An Immune Basis for Lung Parenchymal Destruction in Chronic Obstructive Pulmonary Disease and Emphysema

**DOI:** 10.1371/journal.pmed.0010008

**Published:** 2004-10-19

**Authors:** Sandra Grumelli, David B Corry, Li-Zhen Song, Ling Song, Linda Green, Joseph Huh, Joan Hacken, Rafael Espada, Remzi Bag, Dorothy E Lewis, Farrah Kheradmand

**Affiliations:** 1Department of Medicine, Section of Pulmonary and Critical Care, Baylor College of MedicineHouston, TexasUnited States of America; **2**Department of Immunology, Baylor College of MedicineHouston, TexasUnited States of America; **3**Department of Pathology, Michael E. DeBakey Veterans Affairs Medical CenterHouston, TexasUnited States of America; **4**Department of Surgery, Michael E. DeBakey Veterans Affairs Medical CenterHouston, TexasUnited States of America; **5**Department of Radiology, Michael E. DeBakey Veterans Affairs Medical CenterHouston, TexasUnited States of America; **6**Department of Surgery, Baylor College of MedicineHouston, TexasUnited States of America; National Heart and Lung InstituteUnited Kingdom

## Abstract

**Background:**

Chronic obstructive pulmonary disease and emphysema are a frequent result of long-term smoking, but the exact mechanisms, specifically which types of cells are associated with the lung destruction, are unclear.

**Methods and Findings:**

We studied different subsets of lymphocytes taken from portions of human lungs removed surgically to find out which lymphocytes were the most frequent, which cell-surface markers these lymphocytes expressed, and whether the lymphocytes secreted any specific factors that could be associated with disease. We found that loss of lung function in patients with chronic obstructive pulmonary disease and emphysema was associated with a high percentage of CD4^+^ and CD8^+^ T lymphocytes that expressed chemokine receptors CCR5 and CXCR3 (both markers of T helper 1 cells), but not CCR3 or CCR4 (markers of T helper 2 cells). Lung lymphocytes in patients with chronic obstructive pulmonary disease and emphysema secrete more interferon gamma—often associated with T helper 1 cells—and interferon-inducible protein 10 and monokine induced by interferon, both of which bind to CXCR3 and are involved in attracting T helper 1 cells. In response to interferon-inducible protein 10 and monokine induced by interferon, but not interferon gamma, lung macrophages secreted macrophage metalloelastase (matrix metalloproteinase-12), a potent elastin-degrading enzyme that causes tissue destruction and which has been linked to emphysema.

**Conclusions:**

These data suggest that Th1 lymphoctytes in the lungs of people with smoking-related damage drive progression of emphysema through CXCR3 ligands, interferon-inducible protein 10, and monokine induced by interferon.

## Abstract 2

The underlying changes that cause smoking-related damage are unclear. This paper suggests that there is a skew towards increased T helper 1 lymphocytes, which may drive progression of this damage

## Introduction

Chronic inhalation of tobacco smoke causes progressive lung destruction in susceptible individuals, resulting in chronic obstructive pulmonary disease (COPD) and emphysema, two well-described clinical syndromes with poorly understood pathogenesis [1,2,3]. A role for T helper cells in the pathogenesis of obstructive lung disease has been established with asthma, where T helper 2 (Th2) cells are strongly linked to both human and experimental disease [4,5,6,7]. A potential role for T cells in COPD has also been suggested in several recent studies that show CD8^+^ T cells are increased in the lungs of people who smoke [8,9,10,11]. T cells cause tissue injury through their secreted products such as cytokines; in mice, overexpression of interleukin (IL)-13, a T cell cytokine that is strongly implicated in the pathogenesis of experimental asthma, resulted in increased production of proteases and enlargement of airspaces reminiscent of emphysema [12]. Further, airway limitation, another characteristic of human asthma, is clinically linked to an accelerated rate of loss of lung function in smoker individuals [13]. It has been suggested, therefore, that asthma and COPD may involve the same type of recruited inflammatory cells, differing only in their location within the lung [14].

Chemokines, their receptors, and cell adhesion molecules regulate migration of immune cells into inflamed tissue [15,16,17,18]. T helper 1 (Th1) cells have been shown to secrete interleukin 2 and interferon gamma (IFN-γ), and express a distinct repertoire of chemokine receptors such as CCR5 and CXCR3 [19,20,21]. In contrast, Th2 cells that are biased to produce IL-4 and IL-5 express mainly CCR4 and CCR3 [22,23,24,25]. Immunofluorescent analysis of airway mucosal biopsies in patients with asthma showed that most T cells co-express IL-4 and CCR4, but, in contrast, T cells in airways of patients with COPD and pulmonary sarcoidosis produce IFN-γ and express high levels of CXCR3, while lacking CCR4 expression [26]. In addition to T cells, a wide variety of other inflammatory cells have been shown to express distinct chemokine receptors that are critical for their homing, suggesting a universal mechanism for regulating immune responses. Interferon-inducible protein 10 (IP-10), monokine induced by interferon gamma (MIG), and interferon-inducible T cell alpha chemoattractant (I-TAC) are three known ligands for CXCR3 produced by normal and injured epithelial cells and T cells that are required for homing of Th1 cells [27,28,29]. In addition to regulation of chemotaxis and homing, other functions have been ascribed to chemokines, including modulation of T cell fate by direct effects on differentiating T cells, and regulation of proteolysis in blood monocytes [19,30].

In this study we determined the dominant T helper phenotype in lung samples from ex-smoker individuals with moderate to severe COPD and emphysema and control individuals with no evidence of smoking-related lung disease. Analysis of chemokine receptor expression on isolated peripheral lung lymphocytes from ex-smokers with COPD/emphysema indicated that both CD4 and CD8 T helper cells are strongly polarized to the Th1 phenotype compared to T cells isolated from lung tissue of normal individuals or individuals with non-smoking-related obstructive lung disease. The same cells spontaneously secreted more IFN-γ and CXCR3 receptor ligands MIG and IP-10 in the COPD and emphysema group than in the group without emphysema. Further, IP-10 and MIG, but not IFN-γ, upregulated macrophage metalloelastase (matrix metalloproteinase [MMP]-12) from isolated lung macrophages. Together, our findings reveal the strong association between COPD/emphysema- and Th1-driven adaptive immunity, suggesting a link to lung destruction mediated by IFN-γ, MIG, and IP-10.

## Methods

### Participants

Twenty-eight non-atopic ex-smoker individuals (see Table 1) undergoing medically necessary lung resection were serially entered into the study: ten individuals with no COPD and no evidence of emphysema (control group) and eighteen individuals (diseased group) with moderate to severe COPD and evidence of emphysema as determined by pulmonary function tests, high-resolution computed tomography (CT), or conventional CT scan. All participants were ex-smokers who had quit smoking for a mean (SD) of 7 (2) y and 4 (2) y in COPD/emphysema and control groups, respectively. COPD was diagnosed according to the criteria recommended by the National Institutes of Health/World Health Organization workshop summary [31]. Participants in the control and COPD/emphysema groups had similar (mean [SD] of 54 [6] and 45 [5], respectively) “pack-year” smoking histories, where smoking one pack of cigarettes per day each year is defined as one pack-year.

**Table 1:**
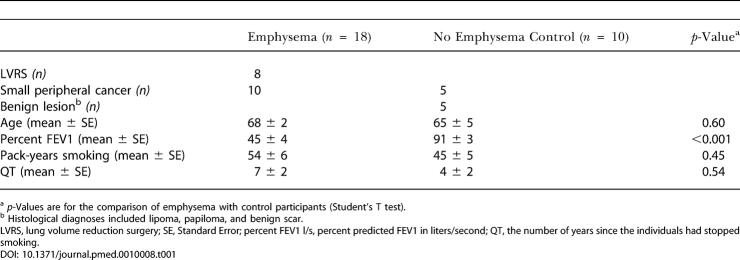
Clinical and Demographic Characteristics of Participants

^a^ *p*-Values are for the comparison of emphysema with control participants (Student's T test)

^b^ Histological diagnoses included lipoma, papiloma, and benign scar

LVRS, lung volume reduction surgery; SE, Standard Error; percent FEV1 l/s, percent predicted FEV1 in liters/second; QT, the number of years since the individuals had stopped smoking

All participants were recruited from the surgical clinic at the Michael E. DeBakey Veterans Affairs Medical Center and the Methodist Hospital, and were undergoing lung resection for diagnostic or therapeutic purposes (Table 1). Study protocols were approved by the institutional review board for human studies, and informed consent was obtained from all participants. Participants had no history of allergy or asthma and had not received oral/systemic corticosteroids during the last 6 mo. At the time of study, all participants had been free of acute symptoms suggestive of upper or lower respiratory tract infection for the 6 wk preceding the study.

### CT-Based Evaluation for Emphysema

High-resolution CT (two in emphysema group and two in control group) or conventional CT analysis was used to detect emphysema, characterized by the presence of areas of low attenuation contrasted with surrounding normal lung parenchyma [32,33]. CT scans were used by a radiologist to separate participants on the basis of the presence or the absence of any objective evidence for centrilobular, panacinar, or paraseptal emphysema with a detection limit of greater than 3-mm low attenuation density [34].

### Isolation of Lung Lymphocytes

Lung lymphocytes were isolated by modifying established protocols, using a combination of mechanical fragmentation, enzyme digestion, and centrifugation procedures described previously [35,36,37]. Viable lymphocytes were separated from whole lung inflammatory cells (macrophages, eosinophils, and neutrophils) using an immunomagnetic positive separation technique (autoMACS, Miltenyi Biotec, Auburn, California, United States). Briefly, lung leukocytes were labeled with paramagnetic bead-conjugated anti-CD3, -CD19, and -CD56 to positively select T, B, and NK cells, according to the manufacturer's instructions. Each of the harvested cell populations was used directly for in vitro assays or was cryopreserved in aliquots of 1 × 10^7^ cells for future analysis.

### Antibodies

The following monoclonal antibodies were purchased from BD Biosciences Pharmingen (San Diego, California, United States): FITC-, Cy5-, and PE-conjugated anti-CD4, -CD8, -CD3, -CD14, -CD69, -CXCR3, -CCR3, -CCR4, and -CCR5. For enzyme-linked immunosorbent assay studies, anti-human antibodies to IFN-γ, IL-4, IP-10, MIG, I-TAC, and the appropriate secondary reagents were purchased from R&D Systems (Minneapolis, Minnesota, United States).

### Quantification of Polarized Peripheral Blood and Lung Lymphocyte Subsets

Phenotypic characterization of T cells was done by two-color flow cytometry (Epic XL FL, Beckman Coulter, Allendale, New Jersey, United States) using combinations of the following monclonal antibodies: FITC-conjugated anti-CD4, -CD8, and -CD14; PE- and Cy5-conjugated anti-CCR4, -CCR3, -CCR5, and -CXCR3. Freshly isolated lung lymphocytes were resuspended to 1 × 10^7^ cells/ml, and 50 μl of cells was incubated with antibodies to CD3 and CD4 or CD8.

### Intracytoplasmic Cytokine Staining

Lung lymphocytes were cultured in the presence or absence of phorbol myristate acetate (PMA)/ionomycin and brefeldin A for 12 h. Cells were harvested, fixed with formaldehyde, permeabilized with saponin, and intracellularly labeled for IFN-γ and IL-4, in addition to staining for surface CD69, CD4, and CD8 according to the manufacturer's recommendations (Fastimmune, BD Biosciences Pharmingen).

### In Vitro T Cell Culture and Cytokine Assay

Lung lymphocytes were isolated from surgical tissue and cultured in vitro in triplicate for 4 d. Supernatants were collected and stored at –80 °C for future analysis. Standard antibody-based enzyme-linked immunosorbent assay was used to measure supernatant concentrations of IP-10, MIG, IL-4, and IFN-γ according to the manufacturer's instructions (R&D Systems and BD Biosciences Pharmingen).

### Detection of MMP12 by Western Blotting, and Real-Time PCR

Peripheral blood mononuclear cells and lung macrophages were isolated by positive selection using immunomagnetic beads conjugated with anti-CD14, and cultured in serum-free medium (RPMI, L-glutamine, and Pen/Strep) prior to overnight stimulation with 0, 50, 250, or 500 ng/ml of IFN-γ, IL-4, MIG, I-TAC, and IP-10. Supernatants were collected, and MMP12 was detected using anti-human MMP12 (R&D Systems) by Western blotting according to the manufacturer's instructions.

Total cellular RNA was extracted from CD14^+^ lung macrophages stimulated overnight with rIP-10 (500 ng/ml) in the presence or absence of blocking anti-CXCR3 antibodies (5 μg/ml, R&D Systems). Two-step real-time reverse transcription PCR was used to determine the relative expression of mRNA using the ABI Perkin Elmer Prism 5700 Sequence Detection System (Applied Biosystems, Foster City, California, United States) as described previously [38].

### Immunostaining and Histopathology

Paraffin-embedded, and fresh-frozen lung sections (5 μm) were immunostained using monoclonal antibodies against human MMP12 (R&D Systems) or non-immune antisera by an immunoperoxidase protocol (Vectastain Elite, Vector Labs, Burlingame, California, United States) and counterstained with hematoxylin as recommended by the manufacturer.

### Statistical Analysis

The Mann-Whitney test (non-parametric, two-tailed) and Student's T test (two-tailed) were used to compare differences between the two groups of subjects. *p* < 0.05 was considered statistically significant.

## Results

### Th1 Immune Bias of Peripheral Lung Lymphocytes in Emphysema

**Figure 1:**
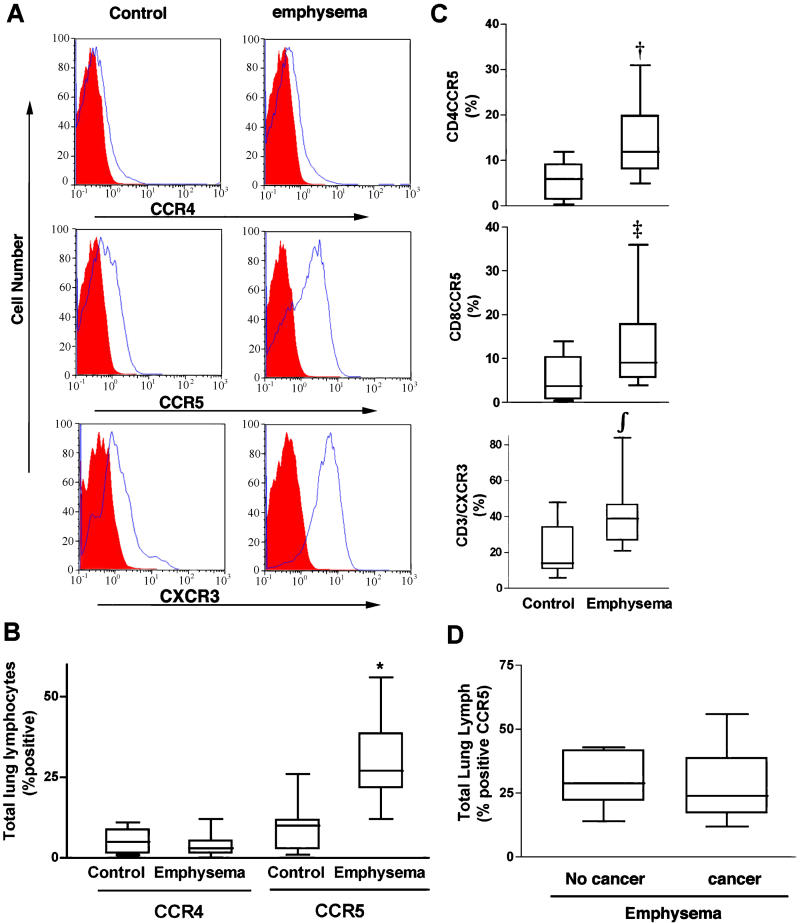
Chemokine Receptor Expression on Peripheral Lung Lymphocytes (A) Single color histograms showing expression of chemokine receptors CCR4, CCR5, and CXCR3 from representative control and emphysema participants. (B) Pooled data from all participants (control, *n =* 10; emphysema, *n =* 18) showing percent (median ± SD) of total lung lymphocytes expressing CCR4 and CCR5. (C) Pooled data from same participants showing percent (median ± SD) CCR5 expression on CD4 (top) and CD8 (middle) T cells, and CXCR3 expression on unfractionated T cells (bottom) from the same participant groups. (D) Analysis of total lung lymphocyte chemokine receptor (median ± SD) profiles among participants with emphysema. Participants had either (1) lung volume reduction surgery for emphysema (non-cancer, *n =* 8) or (2) lung resection for treatment of small peripheral cancer (*n =* 10). Participants showed similar inflammatory indices as determined by CCR5 expression. In (B) and (C), *, *p* < 0.001; †, *p =* 0.01; ‡, *p =* 0.02; ∫, *p =* 0.007 (Mann-Whitney test) for the comparison of emphysema and control groups.

Inflammatory chemokines, cytokines, and their receptors are upregulated at sites of inflammation and play a key role in the recruitment of leukocytes to peripheral tissues in response to injury [17,39]. To detect Th1 polarization, we assessed lung lymphocytes for expression of CCR5 (a receptor for several Th1 chemokines) and CXCR3 (the receptor for IP-10, I-TAC, and MIG). We screened for the presence of Th2 cells by assessing T cell expression of CCR4—a receptor for eotaxin/CCL11, macrophage chemoattractant protein 3 (CCL7), and thymus- and activation-regulated chemokine (CCL17) [40,41]—and CCR3, a receptor for eotaxin and related chemokines. Flow cytometry revealed very low expression of CCR3 and CCR4 (1%–3%) in control (*n =* 10) and emphysema (*n =* 18) groups, and did not discriminate between these populations (Figure 1A and 1B; data not shown). These findings were in sharp contrast to the enhanced expression of both CCR5 and CXCR3, as shown in representative histograms (Figure 1A). These Th1-specific chemokine receptors were expressed prominently on lung lymphocytes from all participants, but their expression was significantly enhanced in the setting of emphysema (Figure 1A–1C). Further, both CD4 and CD8 T cells expressed CCR5 at the same level (Figure 1C). In contrast, we found highly variable expression (0.5%–30%) of CCR4, CXCR3, and CCR5 on peripheral blood lymphocytes isolated from the same participants, and this variation did not correlate with the presence of disease in either group (data not shown). Furthermore, we compared the lung lymphocyte CCR5 and CXCR3 profiles among the eight participants with emphysema alone (lung volume reduction surgery for emphysema; non-cancer) and ten participants with emphysema and accompanying cancer (lung resection for treatment of small peripheral cancer), and found that these two groups cannot be distinguished based on these indices (Figure 1D; data not shown).

**Figure 2:**
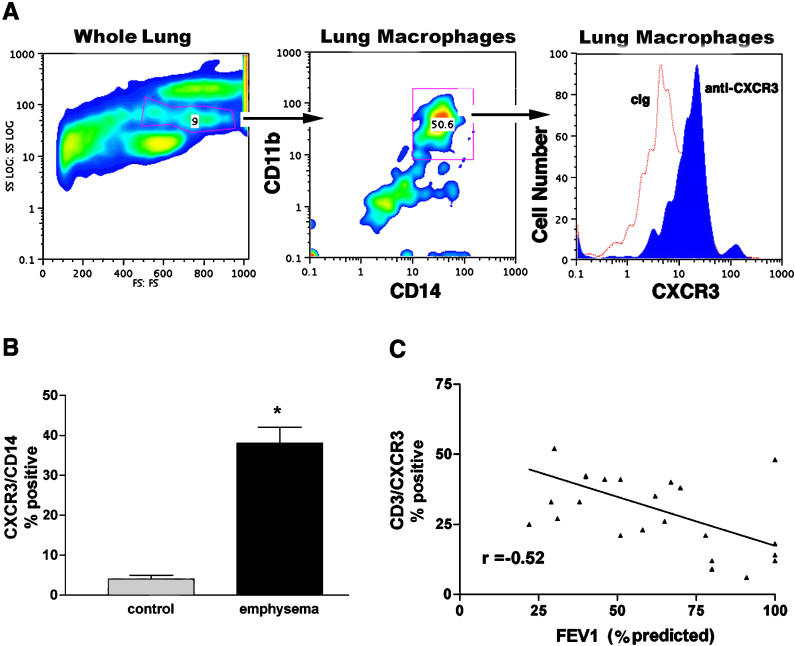
Expression of CXCR3 in Lungs of Control and Emphysematous Smoker Individuals (A) Representative forward and side-scatter characteristics of whole lung cells from a participant with COPD and emphysema. Anti-CD11b PE-conjugated and anti-CD14 FITC-conjugated antibodies detect lung macrophages (middle), and histogram of mean fluorescence intensity showing anti-CXCR3-Cy5 and control antibodies (cIg) detects lung macrophages in the patient with emphysema. (B) Pooled data from control individuals without (*n =* 5) and with (*n =* 8) emphysema. Columns are median, bars represent SD. *, *p =* 0.009 (Mann-Whitney test) for the comparison of emphysema and control participants. (C) Negative association between CXCR3 expression on CD3^+^ T cells and FEV1 percentage predicted based on an *R^2^* goodness-of-fit statistic of 0.27 (*p =* 0.0089, *r* = −0.52, *n =* 24).

Although human lung macrophages are not known to express CXCR3, we suspected based on the immunohistochemical localization of this chemokine receptor that CD14^+^ cells in the lungs of ex-smoker individuals with emphysema accounted for much of the total lung CXCR3^+^ immunoreactivity (Figure 2A; data not shown). To confirm this, we determined the percent of total lung cells expressing CD14 and CD11b—which are both markers of monocytes/macrophages—and CXCR3. We found that over 40% of CD14^+^ cells from participants with emphysema but not control participants were also positive for CXCR3 (Figure 2). In addition, there was a significant negative association between CXCR3 expression on lung T cells and the percent of predicted forced expiratory volume in 1 s (FEV1), based on an *R^2^* goodness-of-fit statistic of 0.27 (Figure 2C; *p* = 0.0089, *r* = −0.52). Together, these data indicate that a strong type 1 bias is characteristic of the T cells isolated from the peripheral lung of participants with COPD and emphysema and that this immune phenotype correlates with the lung destruction that is characteristic of this disease. Further, we have shown for the first time, to our knowledge, that CXCR3 expression, a marker of Th1 inflammation, extends to lung monocytes and macrophages.

### IFN-γ, IP-10, and MIG But Not IL-4 Are Expressed by Lung Lymphocytes

**Figure 3:**
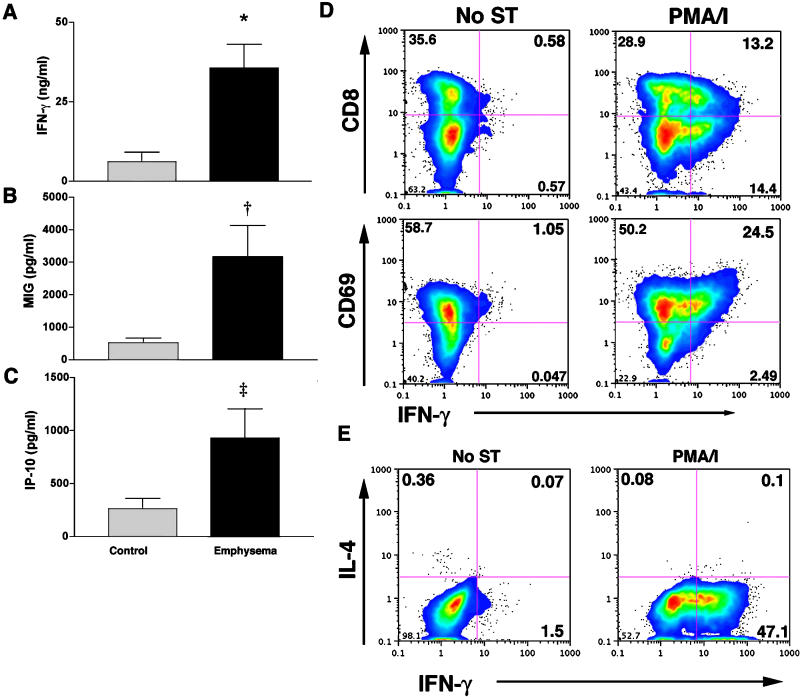
IFN-γ, MIG, and IP-10 Production by Isolated Lung Lymphocytes (A–C) Lung lymphocytes from control individuals and participants with emphysema were cultured without additional stimulation for 3 or 4 d and assessed for secretion of (A) IFN-γ, (B) MIG, and (C) IP-10 (control, *n =* 8; emphysema, *n =* 12). Columns are median, bars represent SD. *, *p=* 0.007; †, *p =* 0.01; ‡, *p =* 0.02 for the comparison of emphysema and control participants. (D) The same cells from a representative ex-smoker individual with emphysema were either left unstimulated (No ST) or treated with PMA/ionomycin (PMA/I) for 24 h and assessed for surface CD8 and CD69 expression and the intracytoplasmic accumulation of IFN-γ by flow cytometry. (E) Production of IL-4 by lung lymphocytes. Lung lymphocytes from a representative ex-smoker individual with emphysema were cultured for 24 h with or without PMA/ionomycin stimulation (PMA/I) and assessed for intracytoplasmic IL-4 and IFN-γ accumulation by flow cytometry.

We sought additional functional data to confirm the apparent Th1 bias of peripheral lung inflammatory cells isolated from ex-smoker individuals. Freshly isolated lung lymphocytes that were not otherwise manipulated secreted high levels of IFN-γ, MIG, and IP-10, with significantly greater secretion of both cytokines from lymphocytes of participants with emphysema (Figure 3A–3C). Interestingly, we could not detect appreciable amounts of I-TAC, another known ligand for CXCR3, in lung lymphocytes of control participants or those with emphysema (data not shown). Similar results were obtained using intracytoplasmic cytokine staining of the same cells (Figure 3D), in which PMA/ionomycin stimulation strongly induced IFN-γ production from CD69^+^/CD8^+^ lung lymphocytes. Surface staining for CD4 was not feasible with this protocol; however, the percentage of CD8^−^/IFN-γ^+^ cells was approximately equal to that of CD8^+^/IFN-γ^+^ cells (median [SD], 19[6] versus 16[4], respectively). Because total numbers of CD4^+^ and CD8^+^ T cells were approximately equivalent, this suggests that non-CD8^+^/IFN-γ^+^ cells are largely CD4^+^, and therefore Th1 cells. Finally, the typical Th2 cytokine, IL-4, was not detected in either group, as determined by enzyme-linked immunosorbent assay or intracytoplasmic cytokine staining (Figure 3E; data not shown), confirming the marked Th1 bias of the immune response that underlies smoking-related lung inflammation and emphysema.

### IP-10 and MIG But Not IFN-γ Directly Upregulate MMP12 through CXCR3

**Figure 4:**
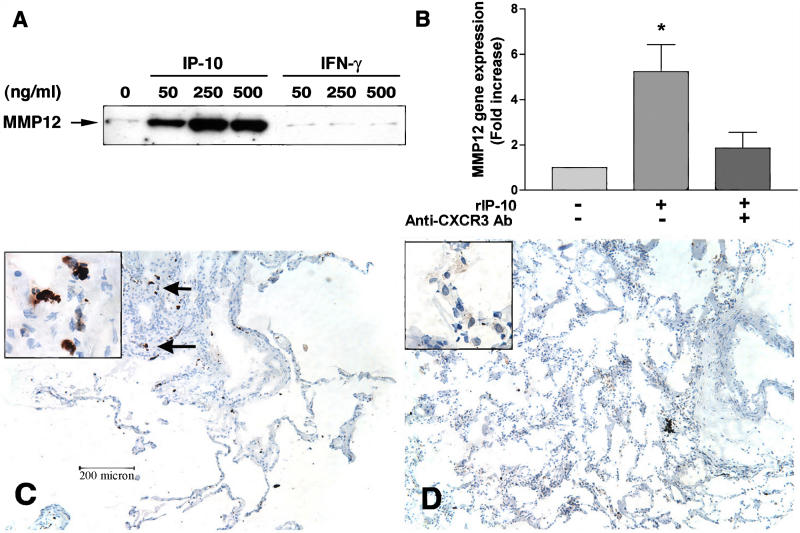
Regulation of MMP12 by Type 1 Cytokines (A) CD14^+^, lymphocyte-depleted lung leukocytes were cultured with and without the indicated amounts of recombinant human IP-10 and IFN-γ, and supernatants were assessed for the presence of MMP12 by Western blotting. (B) Fold increase relative to unstimulated of MMP12 mRNA from lung macrophages stimulated without (**–**) and with (**+**) 500 ng/ml of IP-10 in the presence or absence of a function-blocking antibody to CXCR3 as determined by real-time PCR. (C and D) Lung tissue from a participant with emphysema (C) shows strong immune staining for MMP12 localized to macrophages (arrows), and (D) shows lung tissue from a control participant without emphysema and with undetectable MMP12. The insets show a high-power view of lung macrophages staining positive (C) and negative (D) for MMP12 (×60) *, *p =* 0.04.

Emphysema and irreversible airway limitation that is characteristic of chronic tobacco smoking are related to the destruction of elastin and the resulting loss of lung elastic recoil. Therefore, to be relevant to the pathogenesis of airway obstruction, type 1 inflammation must be shown to promote lung elastolysis. Because loss of elastin is regulated by proteinases [42], we next determined if expression of MMPs, in particular the elastases MMP9 and MMP12, was regulated by IP-10, MIG, and IFN-γ, the principal cytokines detected in emphysematous lung. Indeed, isolated peripheral lung macrophages, but not isolated blood monocytes, secreted MMP12 in response to IP-10 and MIG, but not IFN-γ (Figure 4A; data not shown). These findings reflect a specific receptor–ligand interaction because in the presence of a CXCR3 function-blocking antibody, IP-10 failed to induce MMP12 (Figure 4B). Furthermore, immunohistochemical studies revealed that lung macrophages of participants with emphysema, but not control participants, specifically express MMP12 (Figure 4C and 4D). Together, these findings indicate that Th1, but not Th2, cytokines and related chemokines are required for establishing the pro-elastolytic lung environment that underlies human emphysema.

## Discussion

In this investigation, we characterized T cells and lung macrophages isolated from emphysematous and non-emphysematous human lungs. Three principal findings emerge from our study. First, rather than being functionally diverse, as suggested by the heterogeneous nature of humans, lung T cells of ex-smoker individuals with emphysema are relatively homogeneous and characterized by a marked Th1 bias. Second, the principal Th1 chemokines, MIG and IP-10, are linked to a pro-elastolytic lung environment because these cytokines upregulate the elastase MMP12, which is associated with emphysema. Finally, we found no significant expression of Th2 chemokine receptors, such as CCR3 and CCR4, or IL-4 production in lung lymphocytes. Together, our findings demonstrate the role of the adaptive immune response in COPD and suggest a primary role for Th1 cells in controlling the main smoking-related physiologic and structural changes of the lung.

Upregulation of CCR5 and CXCR3 on T cells and accumulation of these cells in the lung periphery suggest that aberrant, unremitting pulmonary recruitment of these activated T cells is unique to people with smoking-related lung disease, despite cessation of exposure to the inciting agent, tobacco smoke. We showed that ex-smoker individuals without obstructive lung disease or emphysema have comparatively little Th1-biased inflammation in their lungs; thus, our findings reflect the inflammatory changes that are unique to the COPD microenvironment. Additionally, lung lymphocytes isolated from four lifelong non-smoker individuals with severe obstructive lung disease due to cystic fibrosis or bronchiolitis obliterans did not show a Th1 inflammatory bias of the lung (S. Grumelli, F. Kheradmand, D. B. Corry, unpublished data). This information confirmed our finding that the predominant Th1 bias in COPD/emphysema reflects the microenvironment unique to the lungs of ex-smoker individuals. The prevalence of asthma among people who smoke is currently not known, but in order to study COPD/emphysema in a population without other confounding variables, people who might have had asthma were excluded, and thus our findings are restricted to non-asthmatic individuals with emphysema.

Our use of T cell chemokine receptor expression analysis to determine recruitment of lung T cells is not without precedent. Analysis by immunohistochemistry of airway mucosa of people with atopic asthma after antigen challenge revealed that large numbers of CCR4^+^ and CCR8^+^ T cells express IL-4, and CCR4 expression was prominent in people with severe atopic dermatitis, which decreased upon abatement of disease activity [26,43]. Immunostaining of T cells in synovial fluid from individuals with rheumatoid arthritis showed that virtually all of the T cells associated with inflamed joints expressed CXCR3 and CCR5, representing significant enrichment compared to blood T cells from the same participants. Furthermore, previous studies of smoker individuals with COPD and normal lung function showed the presence of CD8^+^/CXCR3^+^ T cells in the airway epithelium and submucosa [44]. We extend these findings by showing CXCR3 expression on lung macrophages and CD4^+^ T cells in emphysema patients and the functional interplay between Th1-related chemokines and elastolytic MMPs.

In addition to detailing surface chemokine receptor expression, we have functionally confirmed the marked Th1 bias of peripheral lung T cells, demonstrating that either at rest or following stimulation, these cells secrete IFN-γ and not IL-4. Our findings therefore confirm the utility of chemokine receptor expression patterns in the initial assessment of T cell effector phenotype.

Destruction of lung parenchyma in emphysema is thought to occur through excessive proteolysis mediated by the elastin-degrading enzymes MMP2, MMP9, and MMP12 from the MMP family, and by neutrophil elastase from the serine proteinase family [45,46]. Cytokines and chemokines are substrates for MMPs, but they also regulate expression of MMPs under pathological conditions [47,48]. We have shown here that IP-10 and MIG, two chemokines that are secreted from lung lymphocytes of participants with emphysema, upregulate specifically MMP12 and thus favor a proteolytic microenvironment that facilitates lung destruction. Strengthening the association between lung macrophages and IP-10/MIG-dependent MMP12 secretion is the fact that we have demonstrated that in humans macrophages, like T cells, express CXCR3 and that this receptor is required for MMP12 secretion in response to IP-10/MIG stimulation. In addition to defining the predominant immune phenotype of emphysematous lung, these additional findings implicate the principal cell (macrophage), MMP (MMP12), and effector cytokines (IFN-γ, IP-10, and MIG) as likely underlying smoking-induced lung destruction. We have further shown that these enzymes may be regulated by proximal immune events driven by Th1 cells or Th1-associated cytokines. A question of major importance for future study is, therefore, the nature of the antigens and adjuvant factors that ultimately drive this inflammatory response.

Although this was an entirely human study, our findings show remarkable parallels with studies performed in mice. MMP12 deficiency has been shown to protect mice against emphysema after chronic exposure to cigarette smoke, implying that MMP12 may be the key proteinase in the development of emphysema in this species [49,50]. Studies from both humans and mice therefore firmly suggest the importance of MMP12 in the pathogenesis of emphysema. Interestingly, in addition to solubilizing elastin, MMP12 is the MMP most efficient at degrading α1-antitrypsin, the primary physiological inhibitor of human leukocyte elastase [51,52]. Thus, chemokine-induced upregulation of MMP12 may orchestrate lung matrix degradation both directly and indirectly through inactivation of α1-antitrypsin. The therapy of COPD and emphysema is currently limited to pharmacologic bronchodilation to relieve dyspnea, antibiotics for intercurrent respiratory tract infection, and vaccination against prominent respiratory pathogens. Aside from efforts to prevent smoking or encourage cessation, there exist no measures that prevent development of emphysema or treat the specific causes of airway obstruction. By providing insight into the immunopathogenesis of COPD, our findings provide genuine hope that future therapies capable of preventing or halting smoking-related lung disease may be possible.

**Patient Summary**

**Background**

Many people develop long-term lung problems after smoking, including a condition called emphysema. At the very end of the airways are tiny air sacs. In healthy people, the air sacs stretch and relax easily on breathing in and out. But in people with emphysema, the air sacs fill up with air but can't empty out properly, so air gets trapped, making breathing difficult. While the symptoms of emphysema can be treated, there are no treatments that can reverse the damage to the lung.

**What Did the Researchers Find?**

The researchers studied two groups of patients, all ex-smokers who had been admitted to a hospital to have part of their lung removed—some because of cancer, some for other reasons. The researchers studied the lung samples and looked to see exactly what type of immune cells the patients with emphysema had in their lungs and found that most of the immune cells were of one particular type. The researchers also showed that the immune cells could tell other lung cells to produce chemicals that can damage the lung.

**What Does This Mean for Patients?**

Lung damage in emphysema may not be caused directly by toxins in cigarette smoke. Instead, if you have emphysema, your body may react to the toxins and produce a special kind of immune cell that is key in causing the lung damage. So perhaps if doctors can find a way to change how this cell behaves, it might be possible to reduce or limit the lung damage. Obviously, not smoking, or stopping smoking, is the best way to prevent COPD or emphysema.

**What Are the Problems with the Study?**

The study is quite small, which means that the results may not be completely accurate; in particular, the study did not include detailed information from patients who had never smoked. So it is too soon to say for sure whether these special immune cells really are the link between smoking and lung damage in emphysema. Researchers will need to study many more patients with emphysema as well as people who have never smoked.

**Where Can I Find More Information?**

Two places to start are the patient Web pages of the following professional organizations.

American Association for Respiratory Care: http://www.yourlunghealth.org/diseases_conditions/copd/

The British Thoracic Society: http://www.brit-thoracic.org.uk/public_content.asp?pageid=9&catid=21&subcatid=177

## Abbreviations

COPD: chronic obstructive pulmonary disease
CT: computed tomography
FEV1: forced expiratory volume in 1 s
IL: interleukin
INF-γ: interferon gamma
IP-10: interferon-inducible protein 10
I-TAC: interferon-inducible T cell alpha chemoattractant
MIG: monokine induced by interferon
MMP: matrix metalloproteinase
PMA: phorbol myristate acetate
Th1: T helper 1
Th2: T helper 2
